# Biodegradation of selected hydrocarbons by novel bacterial strains isolated from contaminated Arabian Gulf sediment

**DOI:** 10.1038/s41598-020-78733-0

**Published:** 2020-12-14

**Authors:** Assad Ahmed Al-Thukair, Karim Malik, Alexis Nzila

**Affiliations:** 1 Life Sciences, King Fahd University of Petroleum and Minerals, Dhahran, Saudi Arabia; 2 Department of Geography and Environmental Studies, Wilfrid Laurier University, Waterloo, Canada

**Keywords:** Microbiology, Environmental sciences

## Abstract

Three strains of novel bacteria were isolated from oil-contaminated sediment from the Arabian Gulf (*Brevibacillus brevis T2C2008*, *Proteus mirabilis T2A12001*, and *Rhodococcus quinshengi TA13008*). The isolated strains were tested for their degrading efficacy of low and high molecular hydrocarbon (naphthalene and pyrene). The efficacy of the two-hydrocarbon degradation by the isolates bacterial was determined at a temperature of 25 °C and 37 °C and pH of 5.0 and 9.0. In inoculated media at 37 °C, *Rhodococcus qinshengi* fully metabolized naphthalene and degrade 56% of pyrene. *Brevibacillus brevis* break down over 80% of naphthalene at room temperatures (25 °C). However, it was found that *P. mirabilis* and *R. qinshengi* biodegraded nearly 94% of naphthalene in the incubated media. The capacity for pyrene and naphthalene degradation in varying pH and temperature conditions was shown to be significant in *Rhodococcus qinshengi* because of its mineralization exceeding 50% across the tested pH and temperature. This implies that the isolated strains are ideal for biodegradation of contaminated sediment with naphthalene and pyrene.

## Introduction

Contamination of the natural environment with oil, including polycyclic aromatic hydrocarbons (PAHs), is a widespread concern because of the health risks associated with these potential cancerous and mutagenic compounds^1,2^. The health risk associated with PAHs is primarily due to their lipophilic nature and inherent tendency to bioaccumulate and eventually undergo bioconcentration and biomagnification in food chains^3,4^.

Microbial degradation potential of oil components seems to decline from: n-alkanes, branched alkanes, monoaromatics, cyclic alkanes, polycyclic aromatic hydrocarbons, and ultimately asphalt and resin^5^. As in most environments, bacteria are the predominant candidates responsible for the natural dissipation of PAHs. *Pseudomonas, Rhodococcus*, *Paenibacillus*, and *Ralstonia* species are among the most intensively investigated bacteria with respect to PAH biodegradation^6–10^. In addition to the bacteria’s active role in biodegradation, a wide variety of yeast and filamentous fungi, algae, cyanobacteria, and some protozoan organisms could degrade different types of hydrocarbons^11–15^. For example, it was reported that pyrene biodegradation mediated by the genera *Rhodococcus* in contaminated environments^16,17^. Wong et al.^18^ in his study highlighted the effects of pH on PAH bioremediation and observed the exponential bacterial growth at pH ranges of 5.5–7.5. In general, most PAHs can get optimal bacterial degradation at pH 7.5^19^. However, several bacterial strains are sensitive to low pH values, which leads to reduced biodegradation^20^. PAH degradation was reported in extremely acidic soils (pH 2)^21^. However, pyrene was shown to biodegrade at pH values above 7.0^22^. Al-Thukair and Malik^23^ have documented improved degradation of pyrene under alkaline medium.

The bioavailability of PAHs at contaminated sites is a critical determinant of the biodegradation kinetics. Naphthalene is the simplest of PAHs and the most soluble and bioavailable hydrocarbon to be degraded by microbes. The ease with which naphthalene is solubilized by micelles in liquid media enhances its bioavailability and biological bacterial attack^24^. *Pseudomonas aeruginosa* for example can make use of naphthalene as its sole source of carbon^25^ and *Streptomyces* sp. is known to grow effectively on naphthalene, using it as the main source of carbon^26^. In addition, *Bacillus fusiformis* can mineralize close to 100% naphthalene within 96 h at 30 °C^27^.

For most hydrocarbon-degrading microbes, the ideal temperature ranges from 25 to 30 °C^28^. Thermophilic bacteria *Bacillus* sp. and *Thermus brockii* efficiently metabolized pyrene and benzo (a) pyrene at 60 °C and 70 °C^29^. Pyrene comprises peri-fused benzene rings, low solubility, and poor bioavailability; hence, it is highly persistent in the environment^30^. It was found that in 20 days, *Pseudomonas* sp. WJ6 could metabolize 19.5% of the provided pyrene. As the initial concentration of pyrene seems to substantially affect the rate and magnitude of biodegradation^28^. For example, when *Mycobacterium* sp. JS19b1 inoculated to an initial concentration of 40 ppm of pyrene, it was able effectively to degrade 100% of the pyrene in 2 weeks^29^. However, optimum pyrene degradation tends to occur above room temperatures^31,32^. In related study carried by Singh et al.^33^ confirmed 60% maximum degradation of pyrene at 37 °C. Al-Thukair and Malik^23^ demonstrated over 50% pyrene degradation by *B. fungorum* at 37 °C.

This study investigates the degradation of naphthalene and pyrene by novel bacterial isolates including; *Brevibacillus brevis* (T2C2008), *Proteus mirabilis* (T2A12001), and *Rhodococcus quinshengi* (TA13008). The three novel bacterial isolates were tested to determine their degrading ability at different temperatures and pH values. The selected temperatures (25 °C, 37 °C) and pH (5, 9) were used to test the hypothesis that the degradation rate is influenced by temperatures and pH. As there is no available literature to the best of the authors’ knowledge describing the biodegradation of naphthalene and pyrene involving bacterial isolates from the Arabian Gulf. Therefore, it is of interest to assess the efficiency of PAH degradation of the isolated strains and the optimal conditions for their biodegradation. This study will contribute to identifying competent bacteria isolates for utilization in the clean-up process of hydrocarbon-polluted sites in the future.

## Material and methods

### Materials and microorganisms

Previously isolated from oil-contaminated sites, the bacterial strains *Brevibacillus brevis*, *Proteus mirabilis*, and *Rhodoccus quinshengi* were identified via 16S RNA. The above isolates were described, identified, and preserved in previous research carried out at the Life Sciences Department of the King Fahd University of Petroleum and Minerals^23,44^. Pre-culture of the strains in nutrient agar (yeast extract, 2.0 g/L; meat extract, 1.0 g/L; peptone, 5 g/L; NaCl, 5.0 g/L; and agar, 15 g/L) naphthalene and pyrene were of scientific quality (99%). The following ingredients (g/L) were used in Bushnell–Haas mineral medium (BH): MgSO4, 0.2; CaCl2, 0.02; KH2PO4, 1.0; K2HPO4, 1.0; NH4NO3, 1.0 and FeCl3, 0.05. Using phosphate-buffered solution the pH was modified to approximately 7.0 ± 0.2. By autoclaving at 121 °C. The culture media were sterilized for 15 min to remove the biotic agents' propensity to biodegrade the PAHs^23,44^.

### Biodegradation experiment

We focused mainly on two physical parameters (temperature and pH) in our experimental design. In comparison to those observed in our sampling site, two temperatures (37 °C and 25 °C) and pH (5.0 and 9.0) values were chosen. At mesothermic and room temperatures (37 °C and 25 °C), the cultures mixed with naphthalene and pyrene were incubated. Media pH was set to 5.0 and 9.0, and the samples had been incubated. Erlenmeyer flasks (250 mL) held liquid media (BH) with mineral salts of 100 mL for incubation at the temperatures and pH values selected. The flask furnished with 1 mL of naphthalene and pyrene, and 2 ml of inoculum suspension; thus, the initial hydrocarbon concentration in each culture flask was 100 ppm. For 18 days in shake-incubators, flasks were incubated using a Wise Cube Fuzzy System (WIS-20 model) with continuous shaking at 120 rpm. The system included a control and two replicates. The autoclaved bacterial cultures constituted monitors to compensate for the hydrocarbon abiotic loss^23,44^.

### Sample extraction and preparation

Residual pyrene and naphthalene analyses were performed to determine the degradation potentials of each bacterial isolate in specified intervals of 3 days. Solid-phase micro-extraction (SPME) using poly (dimethylsiloxane)-coated fibers (PDMS) was used for sample extraction and preparation before the residual PAH analysis. SPME is an equilibrium extraction technique where fibers are immersed in the sample, and target analytes are extracted from aqueous samples as the analytes partition between the aqueous matrix and the fiber coating^34^. The SPME instrument was soaked with agitation in the samples and allowed to stabilize for 30 min^35^.

### GC–MS analysis

Residual pyrene and naphthalene analysis and detection are performed using gas chromatographic-mass spectrometry (GC/MS). The requirements of the GC device model are as follows: Agilent Technologies (6890N series), Injector Unit (7683B series), and MS System; Inert XL EI/CIMSD (5975B series). The SPME fiber was inserted into the hot injection port after the extraction of residual PAHs that achieved equilibrium where the analytes were desorbed into the GC column. King et al.^35^ adapted the GC/MS system, with slight modifications. An improved analyte removal from the PDMS-coated fiber was used in a desorption time of 10 min. After successive extractions, the fiber was conditioned (blank desorption after each extraction) to restrict analyte carryover effects. The temperature system for GC/MS was as follows: inlet temperature set at 220 °C to maximize desorption, oven temperature set at 50 °C, and ramped at 15 °C/min to 100 °C, kept for 2 min, ramped at 7.5 °C/min to 200 °C and gradually increased to 300 °C.

### Light and scanning electron microscope (SEM)

Morphologies of cellular and colony structures on culture plates and prepared slides were examined using light and scanning microscopes (SEM). Preparation and examination of bacterial samples for SEM have followed the procedure described by Al-Thukair and Malik^23^. The prepared slides were coated with a thin layer of gold and examined in JEOL JSM-6460LV.

### Statistical analysis

Sigma Plot software 11.1 was used to analyze the experimental data collected from biodegradation over the 18-day incubation period. One-way variance analysis (ANOVA) was used to a value of 0.05.

## Results and discussion

### SEM bacterial strains analysis

**Figure 1:**
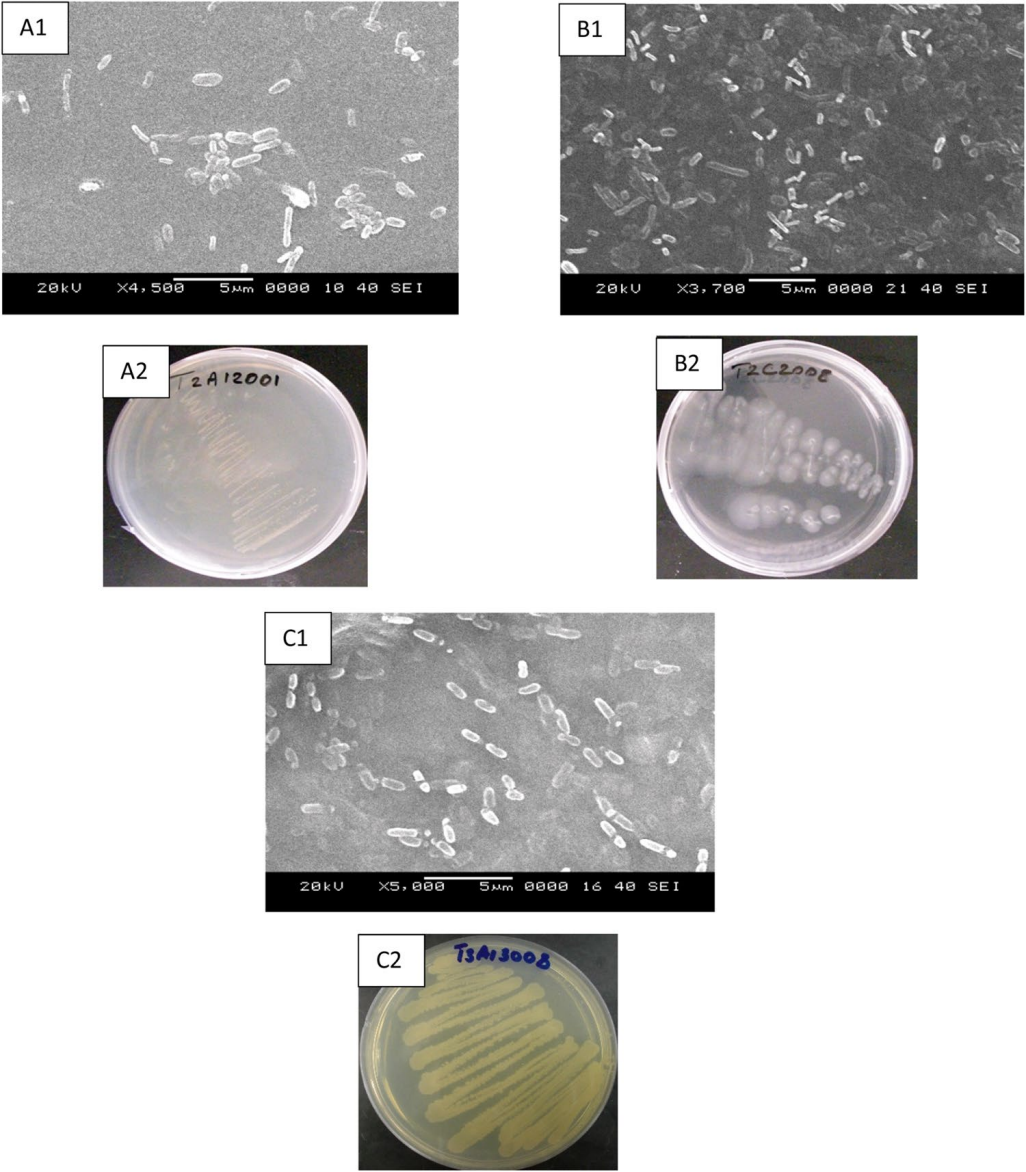
SEM images and photographs of bacteria on nutrient agar plates of *P. mirabilis* (**A1**,**A2**), *B. brevis* (**B1**,**B2**) and *R. quinshengi* (**C1**,**C2**).

Scanning electron microscopy (SEM) was conducted to differentiate the various types of bacterial cells. The SEM allowed to create and confirm the existence of bacteria in the media in which they were cultivated. For growing strain, the strains were morphologically classified using light microscopy as rod forms on nutrient agar plates with varying cell sizes. SEM images showed clearly that the strains of bacteria were rod-shaped with various cell sizes (Fig. 1A1,A2,B1,B2,C1,C2).

### Assessing existing PAHs

**Figure 2:**
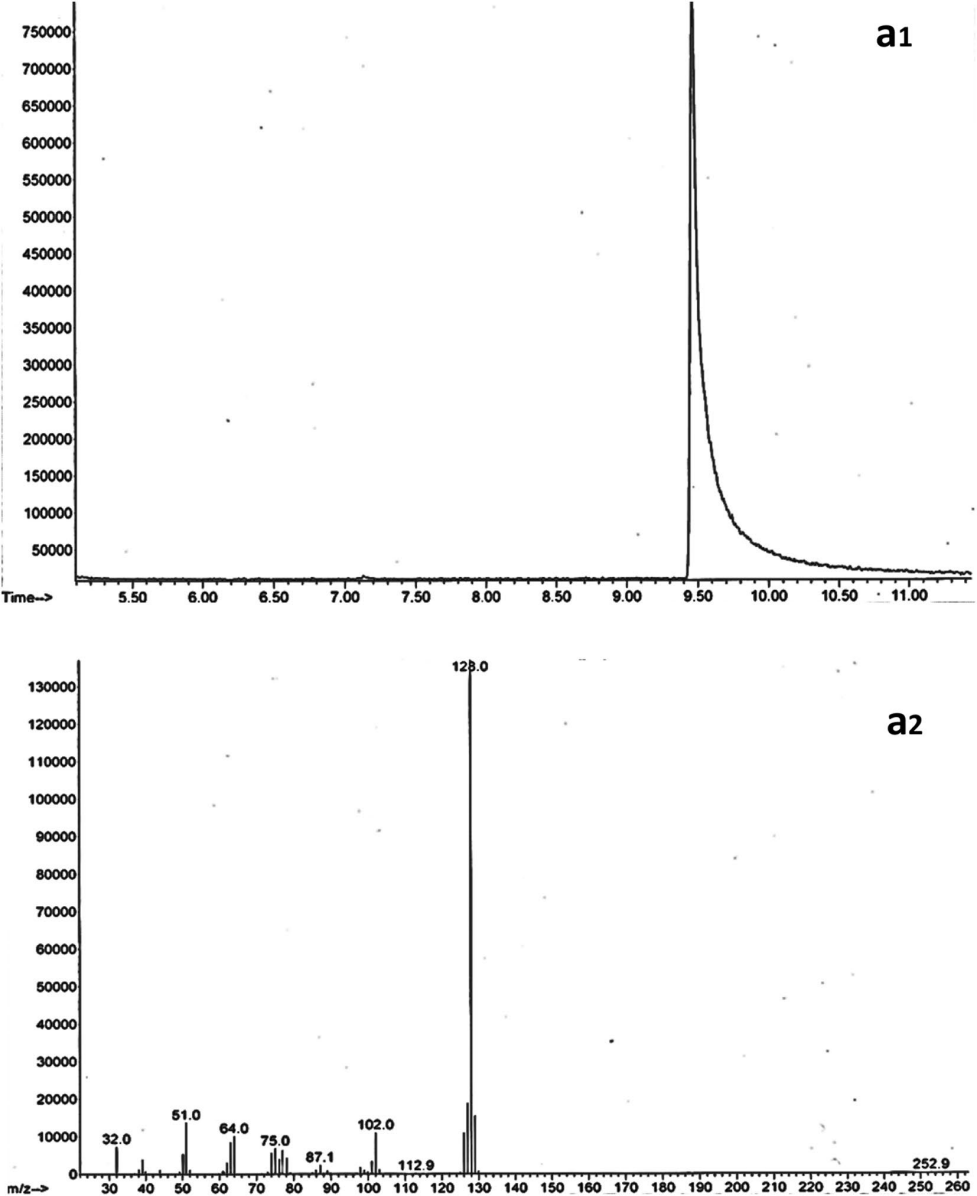
GC Chromatogram (**a1**) and mass spectra (**a2**) of naphthalene.

**Figure 3:**
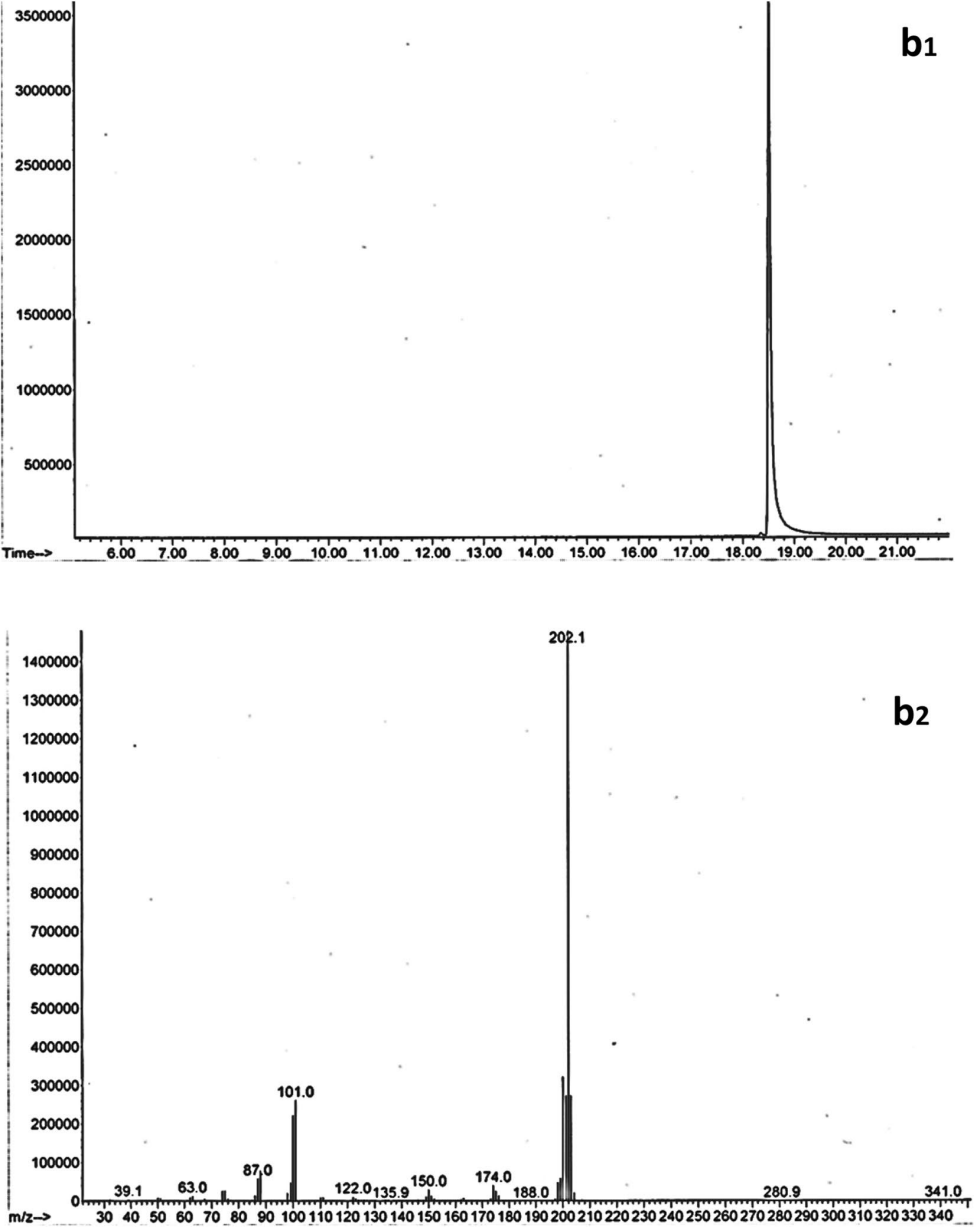
GC Chromatogram (**b1**) and mass spectra (**b2**) of pyrene.

At the end of the biodegradation trials, we subjected samples spiked with naphthalene and pyrene to GC/MS analysis. Figures 2 and 3 show GC chromatograms and naphthalene and pyrene mass spectra, respectively. GC pyrene elution time was estimated to be 18.51 min. The molecular ion had 202.1 m/z, while the fragment ions had 101.0 and 174.0 m/z. Naphthalene was estimated at 9.53 min retention time; molecular ion was at 128.0 m/z, with large fragment ions at 102.0 and 64.0 m/z values.

### Effects of temperature on microbial degradation

#### Naphthalene biodegradation

**Figure 4:**
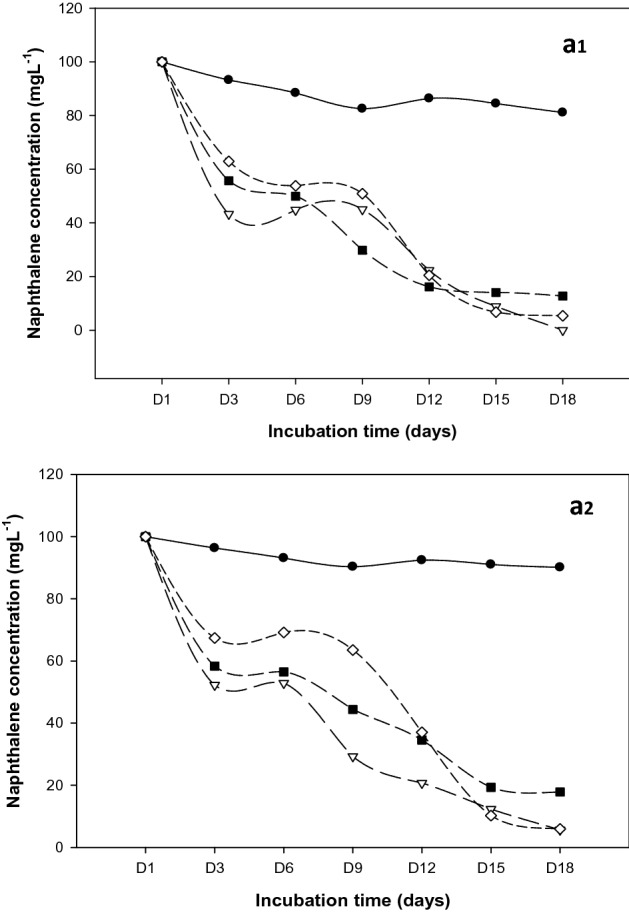
Naphthalene degradation curves at (**a1**) 37 °C and (**a2**) 25 °C; Control experiments (solid sphere), *B. brevis* (solid squares), *P. mirabilis* (diamond), and *R. qinshengi* (triangle).

As simpler PAH is known, naphthalene has substantial solubility and bioavailability with 3–5 fused benzene rings in comparison to other PAHs. Naphthalene was undetected at 18 days of incubation under 37 °C for flasks inoculated with *R. qinshengi* (Fig. 4a1). One logical inference is that biodegradation occurred at 100%. In previous work the low-molecular-weight metabolism of naphthalene by *Geobacillus* strain was realized to be completely degraded in 72 h^36–38^.

**Figure 5:**
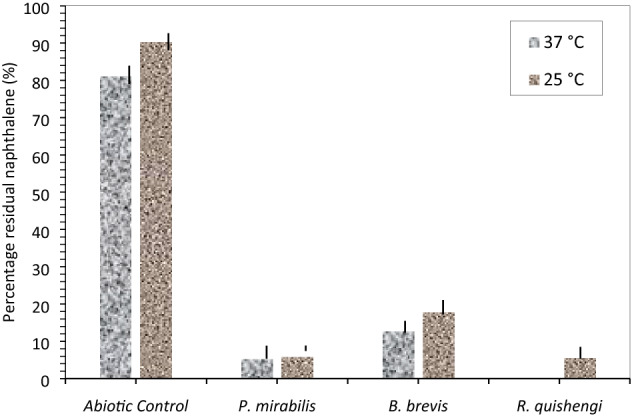
Residual naphthalene in cultures at 37 °C and 25 °C after 18 days, vertical bar represents error bar.

The *P. mirabilis* strain seemed to have biodegraded naphthalene by over 95%. Nevertheless, *B. brevis* decomposed the lowest rates, dissipating 87% of naphthalene. Bacterial metabolism of naphthalene did not decrease significantly at room temperature (25 °C) (Fig. 4a2). For the strains tested, naphthalene was degraded by more than 80% (Fig. 5). Interestingly, *P. mirabilis* and *R qinsheng* had the highest mineralization observed in the inoculated flasks (5.95 ± 0.37 and 5.63 ± 0.35). The strains biodegraded naphthalene by no less than 94%. A comparison of residual naphthalene in the abiotic controls, *P. mirabilis*, *B. brevis*, and *R. quinshengi* is shown in Fig. 5. Efficient naphthalene mineralization at near-room temperatures was highlighted in several studies, such as *Bacillus *sp. SBER3 which biodegrade 75.1% naphthalene at 27 °C^39,40^_._ Under mesothermal conditions, compared with biodegradation, *B. brevis* remain the strain with reduced metabolic activity. Table 1 summarizes the amounts of available naphthalene found in bacterial cultures and control.

**Table 1:** Levels of unmetabolized naphthalene (ppm) in bacterial cultures at day 18 with varying temperatures.

Bacterial strains	Temperature (37 °C)	Temperature (25 °C)
	Mean ± SD	Mean ± SD
*R. quinshengi*	ND	5.63 ± 0.35
*P. mirabilis*	5.4 ± 0.15	5.95 ± 0.37
*B. brevis*	12.73 ± 0.42	17.84 ± 0.89
Abiotic control	81.05 ± 0.91	90.12 ± 0.85

*ND* not detected, *SD* standard deviation.

#### Pyrene biodegradation

**Figure 6:**
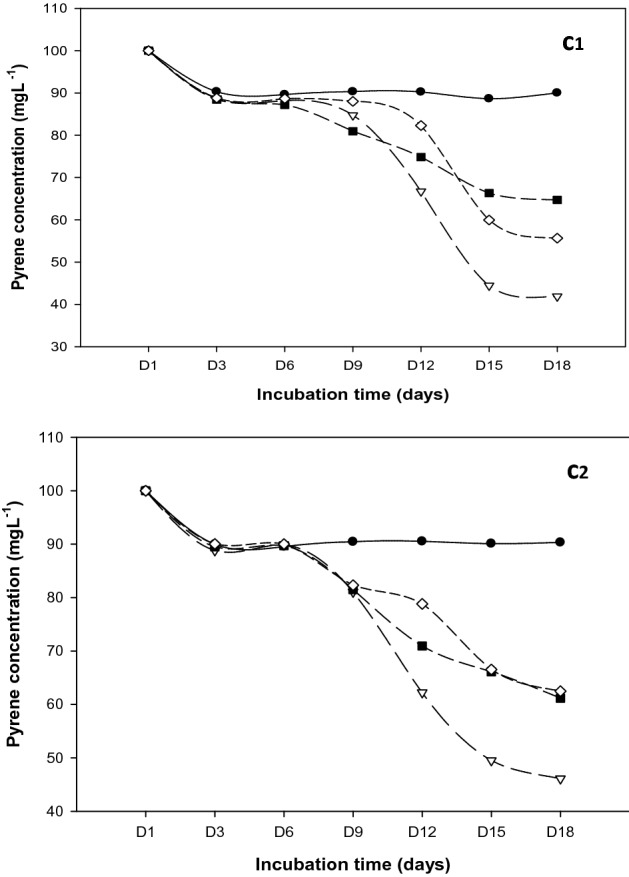
Pyrene degradation curves at (**c1**)37 °C and (**c2**)25 °C; Control experiments (solid sphere), *B. brevis* (solid squares), *P. mirabilis* (diamond), and *R. qinshengi* (triangle).

**Figure 7:**
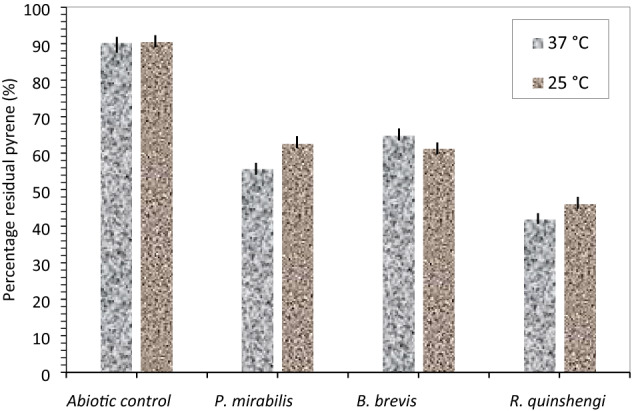
Residual pyrene for the 3 culture strains at 37 °C and 25 °C after 18 days, vertical bar represents error bar.

Because of its comparatively low solubility and bioavailability, pyrene, containing four bonded benzene rings, is extraordinarily resistant to degradation. Few bacteria can metabolize this hydrocarbon altogether. However, pyrene biodegradation has been documented in oil-polluted environments^7,8^. Degradation of the pyrene at 37 °C is shown in Fig. 6c1 and Table 2. Nearly 52% of pyrene was removed by *R. qinshengi* at 25 °C and 37 °C. The level of unmetabolized pyrene measured (Mean ± SD) at 25 °C and 37 °C were 46.09 ± 0.62 and 41.88 ± 0.23 respectively*.* It was found that *Mobacterium sp*. KR2 capable to mineralized nearly 65% of pyrene (at an initial concentration of 500 ppm) in 8 days^41^. Singh et al.^33^ observed a cumulative degradation of 60% at 37 °C in a related experiment. *P. miribilis* biodegraded about the same quantity of hydrocarbon (Fig. 7). The strain biodegrades 44% of pyrene at an initial concentration of 100 ppm. It was found that *B. brevis* strain biodegraded pyrene by less than 36% due to its structural complexity. Xia et al.^30^ reported that *Pseudomonas* sp. WJ6 biodegrade 19.5% of pyrene in 20 days.

**Table 2:** Levels of unmetabolized pyrene (ppm) in bacterial cultures at day 18 with varying temperatures.

Bacterial strains	Temperature (37 °C)	Temperature (25 °C)
	Mean ± SD	Mean ± SD
*R. quinshengi*	41.88 ± 0.23	46.09 ± 0.62
*P. mirabilis*	55.66 ± 0.67	62.47 ± 0.85
*B. brevis*	64.69 ± 1.49	61.16 ± 0.23
Abiotic control	90.01 ± 1.03	90.32 ± 0.97

*SD* standard deviation.

The metabolic activity of the strains has been studied at 25 °C (Fig. 6c2). *P. Mirabilis*, *B. Brevis* biodegrade between 36 and 39%. *B. Brevis* exhibited the least potential for mineralization at 37 °C and 25 °C as levels of unmetabolized pyrene measured at 37 °C and 25 °C were 64.69 ± 1.49 and 61.16 ± 0.23 as shown in Table 2. Figure 7 provides a comparison for the three cultured strains of *P. mirabilis*, *B. Brevis*, and *R. quinshengi* and their percentage of the remaining pyrene in abiotic controls. At an initial concentration of 40 ppm of pyrene, *Mycobacterium* sp., JS19b1 was found to be fully degraded in 14 days^29^. It was also reported that the initial pyrene concentration inhibited the full biodegradation process^42^. Table 2 summarizes the concentrations of unused pyrene found in bacterial cultures and control.

### Effects of pH on microbial biodegradation

#### Naphthalene biodegradation

**Figure 8:**
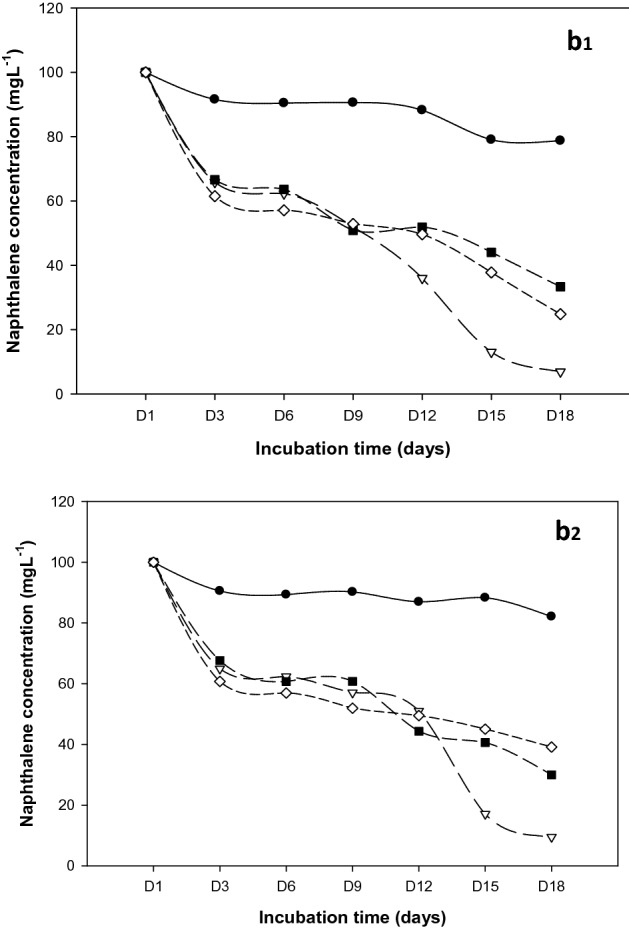
Naphthalene degradation curves at (**b1**) pH 5.0 and (**b2**) pH 9.0; Control experiments (solid sphere), *B. brevis* (solid squares), *P. mirabilis* (diamond), and *R. qinshengi* (triangle).

**Figure 9:**
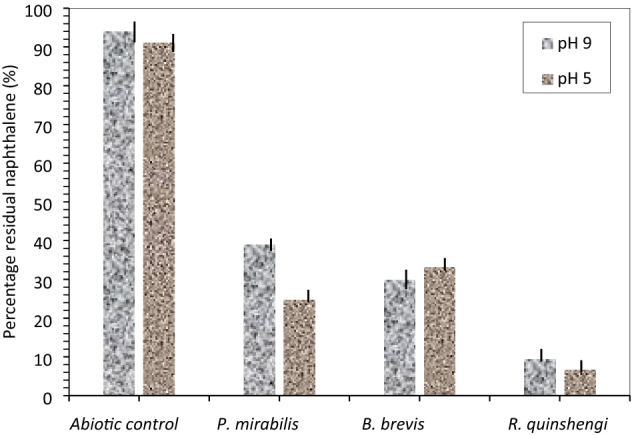
Residual naphthalene in cultures at pH 5.0 and pH 9.0 after 18 days, vertical bar represents error bar.

Biodegradation of naphthalene decreased by many orders of magnitude under acidic conditions (Fig. 8b1, Table 3). However, the highest degradation was reported in *R. qinshengi*-inoculated flasks; in which 93% naphthalene was metabolized by this strain. The mean and standard deviation (Mean ± SD) of residual naphthalene in abiotic control (94.01 ± 0.91), *P. mirabilis* (39.13 ± 1.27), *B. Brevis* (29.99 ± 1.47), and *R. quinshengi* (6.90 ± 0.21) are shown in Fig. 9 and Table 3. It was reported that biodegradation of low-molecular-weight PAH particularly at pH 3–11^37^. Degradation of naphthalene occurred in extremely acidic pH 2 soils with 50% mineralization in 28 days^21^. Microbial metabolism in alkaline samples followed a trend of reduction comparatively like that seen in acidic medium. However, extreme pH is known to inhibit metabolic activity in the bacteria but did not stop biodegradation altogether. The degradation of naphthalene in the alkaline media is shown in Fig. 8b2. The maximum biodegradation of 95.5% occurred in *R. qinshengi*-incubated media. *P. mirabilis* used about 75% of naphthalene quantitatively. Table 3 summarizes the levels of unutilized naphthalene detected in bacterial cultures and abiotic control.

**Table 3:** Levels of unmetabolized naphthalene (ppm) in bacterial cultures at day 18 with varying pH.

Bacterial strains	pH (9.0)	pH (5.0)
	Mean ± SD	Mean ± SD
*R. quinshengi*	9.52 ± 0.73	6.90 ± 0.21
*P. mirabilis*	39.13 ± 1.27	24.81 ± 0.33
*B. brevis*	29.99 ± 1.47	33.31 ± 1.42
Abiotic control	94.01 ± 0.91	91.06 ± 0.85

*SD* standard deviation.

#### Pyrene biodegradation

**Figure 10:**
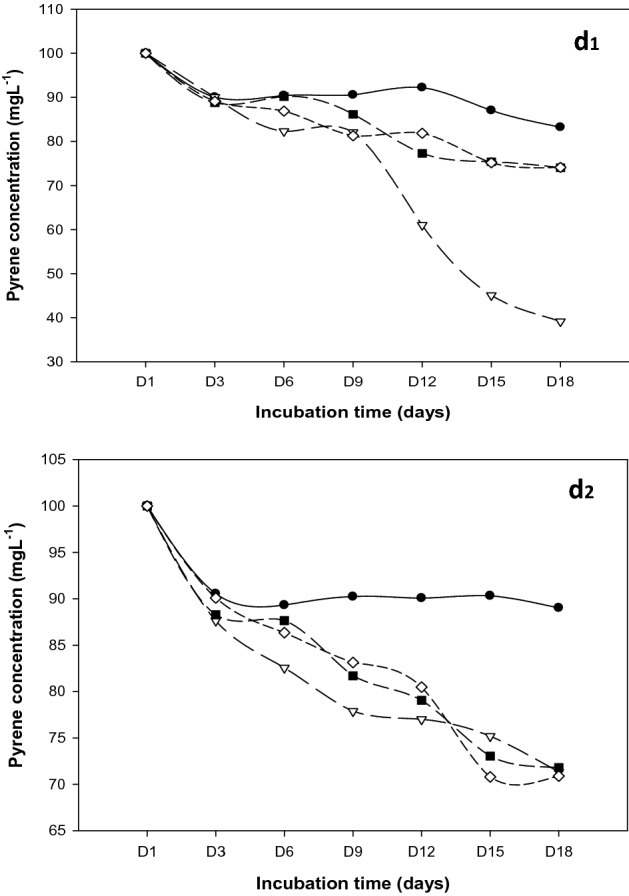
Pyrene degradation curves at (**d1**) pH 5.0 and (**d2**) pH 9.0; Control experiments (solid sphere), *B. brevis* (solid squares), *P. mirabilis* (diamond), and *R. qinshengi* (triangle).

**Figure 11:**
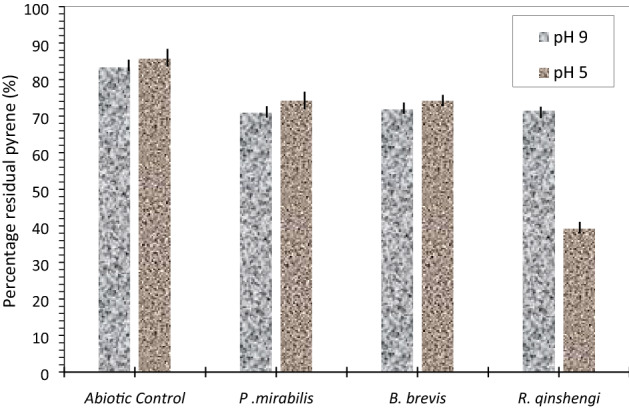
Residual pyrene in culture for the 3 strains at pH 5.0 and pH 9.0 after 18 days, vertical bar represents error bar.

Bacterial metabolism of pyrene appeared to have decreased under acidic medium for all the strains studied (Fig. 10, Table 4). Whereas this statement excludes *R. qinshengi* due to a marginal increase in the metabolic activities of the strain resulting in nearly 61% biodegradation of the initial 100 ppm pyrene. Unlike *P. mirabilis*, and *B. brevis* degraded the pyrene by no less than 26%. In an alkaline medium (pH 9.0), all the strains degraded approximately 30% pyrene. It is essential to reiterate that while biodegradation declined for *R. quinshengi* (71.34 ± 1.53), it increased marginally for *P. mirabilis* (70.89 ± 0.028) and *B. brevis* (71.81 ± 0.40). Increases in the solubility of PAHs by biological-producing bacteria can be due to the comparatively enhanced degradation of alkaline media, which improves biometabolism^42–44^. Figure 11 illustrates a percentage comparison of residual naphthalene in abiotic controls, *P. mirabilis*, *B. Brevis*, and *R. quinshengi*. Table 4 summarizes the concentrations of unused pyrene found in bacterial and abiotic control cultures.

**Table 4:** Levels of unmetabolized pyrene (ppm) in bacterial cultures at day 18 with varying pH.

Bacterial strains	pH (9.0)	pH (5.0)
	Mean ± SD	Mean ± SD
*R. quinshengi*	71.34 ± 1.53	39.16 ± 1.20
*P. mirabilis*	70.89 ± 0.028	74.1 ± 0.49
*B. brevis*	71.81 ± 0.40	74.05 ± 0.86
Abiotic control	83.23 ± 0.93	85.62 ± 1.13

*SD* standard deviation.

## Conclusion

This paper demonstrates that *R. qinshengi* metabolized fully naphthalene and biodegrade 56% of pyrene at mesothermal temperatures (37 °C). *B. Brevis* metabolized naphthalene by more than 80% at room temperature (25 °C). *P. mirabilis* and *R. qinshengi* were noted for the highest metabolism of about 94% of naphthalene. Also, *R. qinshengi* under varying pH conditions, displayed a significant degradation potential for both naphthalene and pyrene. The pyrene mineralization of this strain over the pH spectrum was over 50%. The only exemption was in an alkaline condition where *R. qinshengi* metabolism fell marginally below 30%.

*P. mirabilis*, and *B. Brevis*, are both documented for their biodegradation activity on textile dyes, but not polyaromatic hydrocarbon (PAHs). They are found to actively mediate the degradation of PAHs, especially naphthalene and pyrene. *R. qinshengi* biodegraded naphthalene and pyrene at varying temperatures and pH over the half-life of the initial 100 ppm concentration. Therefore, the three isolated strains are ideal for remediation of contaminated sediment with naphthalene and pyrene.
